# Optical coactivation in cortical cells: reprogramming the excitation-inhibition balancing act to control neuronal gain in abstract and detailed models

**DOI:** 10.1186/1471-2202-15-S1-F1

**Published:** 2014-07-21

**Authors:** Sarah J Jarvis, Konstantin Nikolic, Simon R Schultz

**Affiliations:** 1 Department of Bioengineering, Imperial College, London SW7 2AZ UK; 2 Institute of Biomedical Engineering, Department of Electrical and Electronic Engineering, Imperial College, London SW7 2AZ UK

## 

The interplay of excitatory and inhibitory activity in neuronal populations is finely regulated within cortical layers, with their imbalance being heavily implicated as the underlying cause for many neurological disorders, such as autism, schizophrenia and epilepsy. A key regulatory mechanism is gain modulation, in which the amplitude of response changes while the cell's selectivity remains unaffected. Previous work has addressed gain modulation by examining the interplay of excitatory and inhibitory input at the soma [1]. However, given the non-linear integration that occurs in dendritic arbors, it remains unclear how gain is modulated when the input is located at synaptic locations. For investigating and manipulating this balance of activity throughout the entire neuronal morphology, optogenetics is a powerful tool due to the fine temporal and spatial precision it provides [2]. Furthermore, due to the development of excitatory opsins, such as Channelrhodopsin-2 (ChR2), that depolarize neuronal membrane and silencing opsins, including halorhodopsin (NpHR), that hyperpolarize the membrane, disjoint subdomains of the dendritic and soma morphology can be targeted. This capability has recently been furthered by the development of co-activated opsins, such as ChR2-NpHR [3], which allow independent excitation and inhibition within the same neural population due to the different preferential excitation wavelengths of each opsin (λ=490, 585nm for ChR2 and NpHR respectively). Together, these opsins provide a potential window through which to examine the interplay of competing excitatory and inhibitory inputs for differing spatial and temporal patterns of activation.

We demonstrated previously that gain modulation in a detailed model of a Layer 5 Pyramidal cell using co-activated opsins is possible but highly dependent on the dendritic subdomains targeted [4,5], with whole cell illumination necessary to illicit gain modulation. In contrast, partial illumination of only the apical dendrites and soma resulted in no gain modulation. This suggests a strong link between potential for gain modulation and neuron morphology. While this result helps to untangle the relative contribution of excitatory and inhibitory influences, and warns of inadvertent errors when shallow illumination occurs experimentally.

We investigate this relation by first testing optical activation in abstracted neuron morphologies that include models of ChR2 and NpHR. By creating a family of neural morphologies that extend a simple ball-and-stick neuron model, we investigate how uni-, bi- and multi-polar neurons vary gain modulation upon partial illumination. External driving input is provided as both current injection and as multiple synaptic-like events at locations on dendrites, rather than the soma, to mimic input conditions for both *in vitro* and *in vivo* experiments. Using these models, we identify optimal illumination strategies for each morphological class of neuron, and predict how robust neuronal response is upon partial illumination. Finally, we test detailed neuron morphologies, including stellate interneurons, to test the predictions made by our abstract models.

Our results highlight the role of dendritic subdomains and the localized contribution of excitatory and inhibitory activity in gain modulation. Importantly, our model allows us to predict experimental illumination strategies that are tailored to neuronal morphology and are robust to any limitations that can occur experimentally.
